# Effect of Kidney Volume on the Results of Nephrectomy Performed for Xanthogranulomatous Pyelonephritis

**DOI:** 10.7759/cureus.3976

**Published:** 2019-01-29

**Authors:** Erdem Kisa, Mehmet Zeynel Keskin, Cem Yucel, Okan N Yalbuzdag, Mustafa Karabıcak, Yusuf O Ilbey

**Affiliations:** 1 Urology, University of Health Sciences Tepecik Training and Research Hospital, İzmir, TUR; 2 Urology, University of Health Sciences Tepecik Training and Research Hospital, Izmir, TUR

**Keywords:** xanthogranulomatous, xanthogranulomatous pyelonephritis, nephrectomy, inflammation, kidney tumor

## Abstract

Aim

This study aims to evaluate the results of patients we treated with nephrectomy due to Xanthogranulomatous pyelonephritis (XGP) and the effects of kidney volume on the results.

Patients and methods

Records of 22 patients who underwent nephrectomy due to renal masses at our clinic between January 2008 and May 2018 and whose pathology results indicated XGP were retrospectively evaluated. The computed tomography (CT) measurement of the kidney volumes of the patients was calculated as the product of the longest length, width, and height of the kidney. The mean kidney volume of the patients was calculated and the patients were distributed into two groups: those that presented volumes below average (Group 1) and above average (Group 2). The patients’ mean ages, operative duration, hospitalization days, differences in pre- and postoperative hemoglobin and creatinine levels, and postoperative complications were compared across groups.

Results

Group 1 consisted of 12 patients and Group 2 of 10 patients. The mean kidney volume of the patients was calculated as 33.4 cm^3^ ± 26.0 cm^3^. The mean kidney volume of the patients was 15.8 cm^3^± 9.9 cm^3^ in Group 1 and 56.8 cm^3^ ± 21.8 cm^3^ in Group 2. There were no statistical differences between the two groups in terms of operative times, preop-postop hemoglobin (Hgb) levels and complications.

Conclusion

In cases where XGP is considered probable, the priority in preoperative CT must be to thoroughly evaluate the relationship of the kidney with the surrounding tissue and organs rather than to investigate the patients’ kidney volumes.

## Introduction

First described by Schlagenhaufer in 1916, Xanthogranulomatous pyelonephritis (XGP) is a chronic and serious bacterial infection of the renal parenchyma [1]. Chronic infection usually develops due to the obstruction of the collecting system caused by kidney and ureteral stones [2-3]. The involvement of the renal parenchyma following infection can present a diffused or focal pattern. The diffused form is more prevalent than the focal form [4].

Prepathological diagnosis of XGP can be made when certain findings are present in a computed tomography (CT) scan. Among these findings, the presence of low-attenuation lesions of 10-15 Hounsfield Unit (HU) and minimal or absent contrast in the renal parenchyma are particularly suggestive of XGP [5]. At the same time, CT images may provide insight into the invasion of the renal parenchyma with the surrounding tissues and organs [5].

In cases where XGP is thought to be present due to clinical, laboratory, or imaging findings, the treatment is usually nephrectomy due to the diffuse involvement of the renal parenchyma. Chronic infection of the parenchyma can cause adherence in the renal pelvis, hilum, and the surrounding tissues, as well as the involvement of the neighboring organs. Over time, the obliteration of normal anatomical planes can also be seen [6-7]. This can cause the nephrectomy protocol that will be performed due to XGP to transform into a challenging and life-threatening procedure.

Laparoscopic nephrectomy (LN) has been performed at increasing rates in recent years in the treatment of benign and malign kidney tumors due to providing advantages such as a shorter hospitalization time, less postoperative pain, and early mobilization [8]. We commonly encounter this procedure in case series of laparoscopy performed for XGP in recent years. However, considering the difficulty of nephrectomy that will be performed for suspected XGP, it is emphasized that both the open and laparoscopic approaches must be performed by experienced centers and individuals [9-10].

In this study, we aimed to investigate the clinical properties, difficulties encountered during surgery, and effects of kidney volume on operative values and complications seen in patients who underwent open or laparoscopic nephrectomy due to XGP at our clinic.

## Materials and methods

Pathology reports of patients who underwent nephrectomy at our urology clinic between the January 2008 and May 2018 were retrospectively reviewed. Patients who had XGP-positive pathology results and complete data were included in the study. Patients who had pathology results indicating non-XGP benign and malignant kidney tumours were excluded from the study. For all patients, demographic data, changes during the operation and surgical outcomes were obtained from patient records. Prior to surgery, full blood count results, serum creatinine (Cre) levels, bleeding profiles, and urine cultures of all patients were evaluated. Those who had sterile urine cultures and who had cultures that manifested growth were noted. Those with resistant infections received antibiotherapy before surgery, which extended to the post-surgical period. All patients were evaluated preoperatively with contrast CT and scintigraphy (Tc-99mDMSA). Patients whose imaging results demonstrated calculi were recorded. Comorbidities, American Society of Anesthesiologists (ASA) scores, operative duration, hospitalization days, preoperative-postoperative hemoglobin (Hgb) and Cre levels of the patients were recorded. Postoperative complications encountered in the patients were assessed based on the Dindo-Clavien scoring system [11]. CT measurements of the kidney volumes of the patients were calculated by using the formula for a prolate ellipsoid (maximum bipolar length × maximum width × maximum depth × 0.532). The patients were distributed into two groups: those that presented volumes below average (Group 1) and above average (Group 2). The patients’ mean ages, operative duration, hospitalization days, differences in pre- and postoperative Hgb and Cre levels and postoperative complications were compared across groups. Consent from the ethics committee was not required because of the retrospective nature of this study. Written informed consent to undergo surgery was routinely obtained from each surgical patient.

Patients with XGP-positive pathology results underwent open nephrectomy (ON) and LN. All LN procedures were performed with a transperitoneal approach. None of the LN procedures were converted to ON. ON was done with a transabdominal (anterior subcostal) and retroperitoneal (flank) incision.

Statistical analysis

A Mann-Whitney-U test was performed for the statistical analysis of scale data, and a chi-square test was conducted for ordinal data using the IBM Statistical Package for Social Sciences (SPSS) Version 22.0 program (IBM Corp, Armonk, NY, US).

## Results

Twenty-two patients who had undergone nephrectomy based on CT and scintigraphy results and who had XGP-positive pathology results were included in the study. The preoperative CT scans of the patients provided non-functional images or low-contrast images, suggesting a renal abscess or renal mass. All patients had kidney function values below 10% according to Tc-99mDMSA. The demographic properties of these patients, their history of stone disease, the cause of admission, the cause of nephrectomy, the type of microorganisms that manifested growth in their urine cultures, comorbidities, ASA scores, and operation types have been listed in Table 1.

**Table 1:** The patients’ demographic data SD: Standard Deviation, ASA: American Society of Anesthesiologists

Variables	Overall (n=22)
Age at diagnosis (years)	
Mean ± SD	51±18.3
Median (Min-Max)	53 (3-73)
Gender, No. (%)	
Female	12 (54.5% )
Male	10 (45.4% )
Laterality, No. (%)	
Right	12 (54.5% )
Left	10 (45.4% )
Kidney volume (cm^3^)	
Mean ± SD	33.4 ± 26.0
Nephrolithiasis No. (%)	15/22 (68.1%)
Cause presentation of patients at diagnosis, No. (%)	
Pain	12 (54.5% )
Fewer	6 (27.2% )
Hematuria	2 (9 % )
Asemptomatic	2 (9 % )
Cause of Nephrectomy, No. (%)	
Nonfunctional	14 (63.6%)
Abscess	5 (22.7%)
Renal mass	3 (13.6%)
Preoperative urine culture and organisms found at the time of admission in patients	
Uriner tract enfection	8 (36.3%)
- Escherichiacoli	5 (62.5%)
-Proteus	1 (12.5%)
- Pseudomonas	1 (12.5%)
- S.agalactiae	1 (12.5%)
Steril	14 (63.6%)
Co-morbidities, No. (%)	
None	13 (59 %)
Hypertension	3 (13.6%)
Diabetes Mellitus	6 (27.2%)
Coroner Arteria Disease	3 (13.6%)
ASA score	
ASA 1	1 (4.5%)
ASA 2	15 (68.1%)
ASA 3	5 (22.7%)
ASA 4	1 (4.5%)
Operation	
Open	19/22 (86.3%)
Retroperitoneal (Flank incision)	14/19 (73.6%)
Transabdominal (Chevron incision )	5/19 (26.3%)
Laparoscopic (Transperitoneal)	3/22 (13.6%)

Preoperative and postoperative Hgb and Cre values, operative duration, hospitalization days, and complication rates of the patients have been presented in Table 2. Of the 22 patients who underwent nephrectomy, 19 (86.3%) underwent ON and 3 (13.6%) LN. The mean operative durations were determined as 147.2 min for ON and 193.2 min for LN. Due to the low number of patients in our LN series, statistical comparisons to the ON series could not be performed. With regards to complications, four patients demonstrated Grade 1 complications, three patients needed a blood transfusion (Grade 2 complication), one patient required postoperative antibiotic therapy due to fever (Grade 2 complication), one patient underwent thorax tube insertion due to pneumothorax under local anesthesia (Grade 3 complication), and one patient underwent a splenectomy during the operation (Grade 4 complication) (Table 2).

**Table 2:** The patients’ study results SD: Standard Deviation, Hgb: Hemoglobin, Cre: Creatinine

Variables	Overall (n=22)
Median Hgb Preop-Postop (Min-Max), g/L	1.3 (-2.1-4.3)
Median Cre Preop-Postop (Min-Max), μmol/L	0 ( -0.3-0.2)
Operative time (min)	
Mean ± SD	153.8 ± 44.6
Median (Min-Max)	150 (100-300)
Hospitalization (days)	
Mean ± SD	4±1.4
Median (Min-Max)	4 (2-7)
Complications (Clavien complication rates), n (%)	
Grade1	4/22 (18.1%)
Grade2	4/22 (18.1%)
Grade3	1/22 (4.5%)
Grade4	1/22 (4.5%)
Overall complication rates	10/22 (45.4%)
30 days mortality (%)	0

The average kidney volume of the patients was calculated as 33.4 cm^3^ ± 26.0 cm^3^. The patients were divided into two groups based on this average kidney volume. Group 1 consisted of 12 patients and Group 2 of 10 patients. There were no statistical differences across the two groups in terms of the patients’ mean ages, preop-postop Hgb and Creatinine levels, operative duration, and hospitalization days (Table 3).

**Table 3:** Study results by groups SD: Standard Deviation, Hgb: Hemoglobin, Cre: Creatinine

Variables	Group 1, n=12	Group 2, n=10	p-value
Mean kidney volume ± SD, cm^3^	15.8 ± 9.9	56.8 ± 21.8	
Mean age ± SD, years	49.1 ± 22.9	53.5 ± 10.4	0.72
Mean Hgb Preop-Postop ± SD, g/L	1.41 ± 1.6	1.22 ± 0.73	0.8
Mean Cre Preop-Postop ± SD, μmol/L	0.025 ± 0.12	0.033 ± 0.11	0.31
Mean operative time ± SD, min.	167.5 ± 52.2	135.5 ± 24	0.13
Mean hospitalization ± SD, days	3.75 ± 1.35	4.56 ± 1.59	0.21
Complications (Clavien complication rates), n (%)			0,684
Grade1	2 ( 16.6%)	2 (20%)	
Grade2	2 ( 16.6%)	2 (20%)	
Grade3	0	1 (10%)	
Grade4	1 ( 8.3%)	0	
Overall complication rates	5 ( 41.6%)	5 (50%)	

## Discussion

The literature states that patients whose preoperative imaging results suggest XGP and who undergo nephrectomy could present higher rates of preoperative and postoperative morbidity [6-7]. The CT scans of these patients may demonstrate an invasion of surrounding tissues due to chronic infection. Therefore, organ and vascular injuries, long operative durations, need for intensive care due to complications, and the development of sepsis due to infection must be considered likely, and we think that the patients must be approached with a multidisciplinary plan (urology, general surgery, cardiovascular surgery, anesthesia, and infectious diseases) prior to the operation. Organ injuries, such as colon and liver injuries, have been reported in the literature as case presentations [12-13]. One of the most serious complications encountered in our clinical series was a need for splenectomy during left nephrectomy performed for XGP because the upper pole of the kidney was severely fibrotic and attached to the surrounding tissues. Another case included chest tube insertion under local anesthesia after the pleura was opened during right nephrectomy due to XGP and a diagnosis was made in the postoperative period.

Malek et al. categorized the involvement of the kidney in XGP and in relation to the surrounding tissues under three stages [14]: confined to the kidney (Stage I), involvement of perinephric adipose tissue (Stage II), and involvement of retroperitoneal structures (Stage III). In their series of 17 LN procedures, Rube et al. classified complications according to these stages. Most Grade 3-4 complications, according to the Dindo classification, were seen in kidneys with Stage III XGP [15]. On the other hand, we placed our XGP patients into two groups based on their kidney sizes in the CT scan. Instead of this classification utilized by Malek et al., we calculated kidney sizes based on CT scans and compared those who had kidneys larger than the mean kidney size to those who had kidneys smaller than the mean kidney size. Although large kidneys were expected to be associated with a greater invasion of surrounding tissues and neighbouring organs, no statistical differences were determined between the group with a kidney size below average and the group with a kidney size above average in terms of Clavien complications. This suggests that the relationship of the chronic infectious process with the surrounding tissues and organs is more determinant of preoperative complications than kidney size. The CT scans of patients who are to undergo nephrectomy for XGP must be carefully inspected with regard to the state of the surrounding tissue and organs.

Nephrectomy operations performed due to XGP may pose certain challenges. In our clinical series, difficulties were encountered in cases where nephrectomy was initiated with a retroperitoneal approach that included the opening of the peritoneum because the Gerota fascia was attached to the peritoneum and the opening of the collecting system during dissection performed due to a hydronephrotic kidney. Moreover, there was a discharge of infective material into the region, the conclusion of the procedure with subcapsular nephrectomy, and the appearance of accessory veins. Angeri et al. reported difficulties that diverged from the usual nephrectomy procedure in their series, such as large lymphadenopathies due to the infectious process, non-recognizable renal artery, invasion of surrounding adipose tissue, en bloc clamping for vascular control rather than individual clamping, and the renal vein being incised and controlled primarily [16].

Various approaches and techniques associated with LN performed for the treatment of XGP and infected kidneys have been described. In the series they published, Kapoor et al. reported a success rate of 80% with subcapsular dissection during nephrectomy, particularly in cases of upper pole involvement [17]. In their other publications, they stated that the subcapsular technique could be used in the laparoscopic retroperitoneal approach [18-19]. However, the authors expressed that as the risk of squamous cell cancer due to long-standing calculi in infected kidneys increases with the subcapsular nephrectomy technique, this risk must be taken into consideration [10,20]. Additionally, it has been reported in the literature that the remaining Gerota fascia could result in infected skin fistulas in the future [21]. Another technique being utilized in LN is the combined (both retroperitoneal and transperitoneal) approach, which is particularly preferred in the case of large kidneys and vascular involvement. The aim of this technique is to facilitate retroperitoneal control of the renal artery during LN [22].

It has been reported in the literature that in infected kidneys such as XGP, the operation that was initiated laparoscopically was converted into open surgery due to adherence to surrounding tissues and complications appearing during the procedure [10,20]. While such conversions were more common in the early LN series, they have become less common in recent years [15]. In the three cases we operated, of which one had a pelvic kidney, there have been no conversions to open surgery and all operations were completed laparoscopically. Due to the need to cope with all these complications and the necessity of a multidisciplinary approach, we think that both open and laparoscopic surgery must be performed by experts and at experienced centers in cases where XGP is considered probable.

The adopted surgical approach may be transperitoneal or retroperitoneal. Both of these methods are associated with certain advantages and disadvantages. As known, the transabdominal approach is advantageous, as the entire abdominal cavity is visible and as it allows direct access to main veins such as the vena cava and aorta. However, the release of the hydronephrotic kidney material to the abdomen following damage to the parenchyma may make this procedure disadvantageous. On the other hand, the retroperitoneal (flank) approach poses no risk of transmission of infective material to the abdomen as long as the peritoneum is intact. However, the narrow working area can complicate surgery, which is already difficult due to infection. According to a review of the literature, either approach can be adopted in cases of LN performed for XGP [9-10,18-19]. While we perform LN both transperitoneally and retroperitoneally at our clinic, we prefer the transperitoneal approach in cases of probable XGP, as it allows better evaluation of the surrounding tissues and organs and provides a more comfortable working space. Comparison of operative durations reported in the literature reveals longer operative durations for LN as compared to ON. Similarly, our LN cases presented longer operative durations compared to ON cases, paralleling the literature (193.2 min and 147.2 min, respectively). However, our advancing laparoscopic experience allowed us to perform LN in three of the most recent four cases on whom we operated for XGP.

A consideration of the limitations of this study highlights the retrospective nature of our study, the relatively low number of patients, and the inability to perform statistical analyses comparing LN and ON patients, as our LN patients were in the form of case series, and the involvement of different surgeons in the nephrectomy procedures.

## Conclusions

The characteristics present in clinical, laboratory, and imaging tests have a significant role in the preoperative diagnosis of patients with XGP. In preoperative CT, the priority must be to closely evaluate the relationship of the kidney with the surrounding tissue and organs rather than to investigate kidney volume. When these patients are to undergo nephrectomy, a multidisciplinary approach is required due to the complexity of the surgical process and surgical complications. We conclude that in these cases, both ON and LN must be performed at experienced centers.
